# Patients’ satisfaction with post-operative pain management in Ayder Comprehensive Specialized Hospital, Ethiopia: a cross-sectional study

**DOI:** 10.11604/pamj.2023.45.94.22563

**Published:** 2023-06-21

**Authors:** Niguse Meles Alema, Solomon Weldegebreal Asgedom, Brhane Gebrehiwot Welegebrial, Tesfay Mehari Atey, Ephrem Mebrahtu Araya, Hagazi Gebremedhin, Desalegn Getnet Demsie, Weldu Mammo Werid, Haylay Araya, Abrahaley Mulu, Gebretekle Gebremichael Hailesilase

**Affiliations:** 1 Department of Pharmacy, College of Medicine and Health Sciences, Adigrat University, Adigrat, Ethiopia,; 2 School of Pharmacy, College of Health Sciences, Mekelle University, Mekelle, Ethiopia,; 3 Department of Midwifery, College of Medicine and Health Sciences, Adigrat University, Adigrat, Ethiopia

**Keywords:** Patient satisfaction, pain management, surgical patients, post-operative pain, Ethiopia

## Abstract

**Introduction:**

surgical patients often suffer from inadequate treatment of post-operative pain which potentially results in numerous adverse medical consequences and is a recurring source of patients' dissatisfaction. Thus, this study aimed to investigate patient's satisfaction with their post-operative pain management and its determinants among surgically treated patients in a specialized hospital within Ethiopia.

**Methods:**

an institutional-based prospective cross-sectional study was conducted in the surgical ward of Ayder Comprehensive Specialized Hospital. Data were collected using a semi-structured questionnaire, which was an adoption of the 2010 version of the American Pain Society Patient Outcome Questionnaire, and by reviewing the medical charts of the patients. A stepwise linear regression model was used to analyze the data.

**Results:**

among the 144 patients approached in this study, 112 (77.8%) of them categorized their postoperative pain as moderate to severe. The mean patient satisfaction with their pain management was 7±2.3 on 0-10 numerical rating scale. Despite high levels of pain, the majority of patients (90.3%, n=131) were moderately or completely satisfied with their pain management. Stepwise linear regression analysis found that the determinants of patients' satisfaction were prior chronic pain, prior surgical history, and substance use (F (3,140) = 5.364, adjusted R_2_= 0.084, P=0.02).

**Conclusion:**

the patients were moderately and completely satisfied with their pain management in spite of expressing moderate and severe level of pain intensity. Pain still remains a concern among surgical patients, and effective pain management strategies should be practiced to manage pain and its functional interferences more effectively.

## Introduction

Post-operative pain (POP) is prevalent among surgical patients and is a persistent challenge to the medical community despite efforts to advance pain management protocols in surgical patients [1]. Effective and safe pain management free from unwanted side effects remains a major challenge to the health care professionals [2-5]. Uncontrolled pain can potentially cause delay in discharge as well as physiologic and financial implications to surgical patients [6]. Moreover, inadequate pain management may also result in development of chronic pain [4,5]. The introduction of acute pain teams has improved the overall management of pain in acute settings, most notably in large general hospitals. Nonetheless, pain management is known to be a significant and recurring source of dissatisfaction for patients [3]. On the other hand, adequate pain control enhances the overall patients' comfort and satisfaction, decrease post-operative complications, reduce length of hospital stay [5,7], increase and facilitate rapid recovery of physical activity [6,8], and improves surgical outcome [9]. Attaining adequate post-operative pain management becomes difficult owing to inadequate assessment, poor communication, and individualized experiences and exhibitions of pain. Most patients are satisfied with the pain management they receive even if they suffer from pain [10]. However, patient satisfaction with their pain relief is one measurement outcome of consistent quality improvement and health care process [11,12].

The subjective and complex character of pain presents obstacles to assessment and management by health care professionals, which demands a patient-centered assessment. Limited research has been carried out exploring patient experiences despite the clinical importance of pain management [11,12]. Patients' pain experience varies from patient to patient and from variation in health care staffs. Patients who have been informed about the specifics of their medical treatment and the possible outcomes are generally more satisfied. In contrast, patients who have unrealistic expectations of treatment or outcome tend to be less satisfied than patients with lower or more realistic expectations [5]. The American Pain Society recommends that patients' satisfaction with anti-pain medications taken during hospitalization should be considered as an important indicator of the quality of the health service of the institution. Nowadays, there is a growing concern of healthcare organizations with patients' satisfaction of the health services provided to them. Although, not being the single factor, pain control is a fundamental aspect to evaluate satisfaction with the treatment received during hospitalization [13]. In the study area, there is a paucity of information regarding post-operative pain management in surgical patients. Thus, this study was aimed to assess post-operative patients' satisfaction with their pain management and its determinant factors among surgically treated patients in an Ethiopian context.

## Methods

**Study design and area:** a prospective cross-sectional study was conducted in the surgical ward of Ayder Comprehensive Specialized Hospital (ACSH) which is found in Mekelle city, Northern Ethiopia. It is one of the largest referral and teaching hospital in Northern Ethiopia providing health services for a catchment population of about 10 million people. The hospital has 500 beds with an average of 280 operations being carried out weekly. The hospital did not have any standardized procedures for management of pain and routine pain management protocols were being followed in the hospital.

**Study participants:** the source population was constituted of all patients who underwent surgical procedures in ACSH. All surgically treated patients, who were admitted in the hospital from March 25^th^ to May 25^th^, 2017 and who fulfilled the inclusion criteria, were included in the study. The inclusion criteria were patients who had undergone any type of surgery, who were alert and active to respond, who were 18 years and above. The exclusion criteria were patients who were in critical situation (unable to communicate), patients who underwent emergency surgery, and patients who were admitted to intensive care unit or with known mental health disorders (identified from the patient's medical chart). Moreover, those patients that have been discharged overnight were excluded.

Sample size was computed using a single population proportion sample size estimation formula. For population >10,000, the formula can be provided as n = [(Z1-α/^2^)^2^ p (1-p)]/d^2^, where n= minimum sample size, Z_1-aα/2_ at 95% confidence interval=1.96, p=estimated level of patient satisfaction with POP management (91%) taken from a study conducted in Jimma [14], d= margin of error to be tolerated (0.05). Substituting all in the overhead formula, n=138. Since the total population in our study was <10,000 (2400), the sample can be recalculated using the correction formula as follow, Nf = n/(1+n/N), where, n = minimum sample size (138), Nf = actual sample size using correction formula, N = actual population size (2400). Therefore, substituting all in the above formula, Nf = 131. Considering a 10% of contingency for a non-response rate, the minimum sample size required for this study was 144. All patients who fulfilled the eligibility criteria were enrolled in the study consecutively until the minimum sample size was achieved.

**Data collection method and procedures:** data were collected using a semi-structured questionnaire developed as an adoption of the 2010 version of the American Pain Society Patient Outcome Questionnaire (APSPOQ) [15]. The questionnaire was initially developed in English language then translated to Amharic and Tigrigna and then back-translated to English. Patients' satisfaction levels, attitudes and beliefs about pain and pain management, degree of pain, and influence of pain on functional status were collected via face-to-face interviews.

The revised APSPOQ items are mainly based on an 11-point numerical rating scale (NRS: scores from 0-10). Patients severity of pain (pain intensity) was measured as NRS score which ranges from 0 = “no pain” to 10 = “worst pain possible”. The proportion of time the surgical patient had spent in severe pain after surgery was measured from 0% = “never in severe pain” to 100% = “always in severe pain.” Pain interference was recorded as functional disability due to pain (NRS score from 0 = “did not interfere” to 10 = “completely interfered”) and the effect of pain on mood and emotion like feeling of anxious, depressed, frightened and helpless was measured using NRS score from 0 = “not at all” to 10 = “extremely”. Patient perception of care was measured as the degree of pain relief obtained through combined medicine and non-medicine pain treatment (NRS score from 0% = “no relief” to 100% = “complete relief”). Patient's requests for stronger analgesics and information about their pain treatment options (non-medicine methods or pharmacological methods) were detailed as “yes” or “no.” Degree of satisfaction with the outcomes of pain management was measured as an NRS score from 0 = “extremely dissatisfied” to 10 = “extremely satisfied.”

For the sake of validation and consistency in data collection, the questionnaire was pretested in 10% of patients (i.e., 15 patients) and amendments were made accordingly. A data abstraction checklist was also developed by retrieving reputable literatures to extract relevant information. Patients' satisfaction was the primary outcome of the study and level of pain intensity, socio-demographic characteristics, and substance use were considered as the independent variables. The statistical analysis was carried out using Statistical Package for Social Sciences (SPSS) Version 21 software. First, the data were edited and checked for completeness and consistency then entered into SPSS for analysis. Simple descriptive statistics, including frequencies, percentages, means and standard deviation, were computed to summarize the socio-demographic and clinical characteristics of the study participants. Categorical variables were expressed with frequencies and percentages, while continuous variables were expressed as means ± standard deviations. A stepwise linear regression model was used for analyzing the data. All statistical analyses were performed and a p-value of less than 0.05 was considered as statistically significant.

**Operational definitions: Pain** was defined as an unpleasant sensory and emotional experience associated with actual and potential tissue damage [15]. **Mild pain** was defined as when the American Pain Society Patient Outcome Questionnaire (APSPOQ) score is from 1-3. **Moderate pain** when APSPOQ score is from 4-6 and **severe pain** when APSPOQ score is from 7-10. Participants who score APSPOQ of zero (0) were considered as having **no pain** [16]. **Null, mild, moderate and full patient satisfaction** was defined when APSPOQ pain satisfaction score was 0, 1-4, 5-6 and 7-10 respectively [16].

**Ethical considerations:** ethical clearance was secured from the Institutional Review Board of the College of Health Sciences, Mekelle University. Written consent was obtained from all the study participants after explaining the main aims and purposes of the study. Moreover, the study participants were informed about their full right to terminate the study at any time. Confidentiality and privacy of the participants were also maintained. During the data collection process, data of patients who were at any risk of complications due to pain were shared with their healthcare providers for possible interventions.

## Results

**Socio-demographic characteristics:** a total of 144 patients participated in this study achieving a 100% response rate. The majority (66%) of the study participants were males and the mean age of the patients were 39.1 ± 14.4 years, ranging from 19 to 76 years. Three-quarters of the participants were married and farmers. Almost half (47.9%) of the patients did not have a formal education (Table 1).

**Table 1:** socio-demographic characteristics of patients in Ayder Comprehensive Specialized Hospital from March 25^th^ to May 25^th^, 2017

Variable			N(%)
Sex	Male		95 (66)
	Female		49 (34)
Age(years)	19-34		71 (49.3)
	35-44		27 (18.8)
	45-54		21 (14.6)
	55-64		16 (11.1)
	65 and above		9 (6.3)
Marital status	Married		108 (75)
	Single		33 (22.9)
	Divorced		3 (2.1)
Educational level	No formal education		69 (47.9)
	Primary school(1-8)		20 (13.9)
	Secondary school(9-14)		30 (20.8)
	College and above		25 (17.4)
Occupation	Farmer		62 (43.1)
	Employed		47 (32.6)
	Unemployed		25 (17.4)
	House wife		10 (6.9)
Substance use	Alcohol	Yes	22 (15.27)
		No	122 (84.73)
	Cigarette	Smoker	3 (2.1)
		Ex-smoker	1 (0.7)
		None smoker	140 (97.2)

**Post-operative pain management and management adequacy:** of the total patients recruited to this study, 34 (23.6%) of them received anti-pain medications within 15 minutes of complaint of pain. More than half (58.3%, n=84) of the patients never asked for analgesic during their hospitalization whereas 2 (1.4%) patients never received the analgesic medications they requested. Forty-one patients (28.5%) needed strong medication for their pain relief and 32(22.2%) used non- pharmacological methods to relieve their pain (Table 2).

**Table 2:** processes of pain management of participants in Ayder Comprehensive Specialized Hospital from March 25^th^ to May 25^th^, 2017

Variable	N (%)
**Waiting Time after requesting analgesics (N = 144)**	
Below 15 minute	34(23.6)
Up to 30 minute	11(7.6)
Up to 1 hour	9(6.3)
Beyond 1 hour	4(2.8)
Asked, never received	2(1.4)
Never asked	84(58.3)
**Want stronger medication (N = 144)**	
No	103(71.5)
Yes	41(28.5)
**Use any non-medicine methods to relieve your pain**	
Yes	32 (22.2)
No	112 (77.8)
**Do health care professionals encourage you to use non-pharmacological methods of pain management (N = 144)**	
Never	126(87.5)
Sometimes	16(11.1)
Often	2(1.4)

In this study, the majority of the participants (77.8%) did not use non-pharmacological methods of pain management. Among the total study participants who used non-pharmacological methods of pain management, 43.75%, 12.5%, and 12.5 %, listened to music, used relaxation, and listened to music and prayed to relieve their pain respectively. The majority (92.4%) of the study participants experienced varying degrees of POP within 24 hours of surgical procedures whereas 7.6% of the surgical patients did not experience any sort of pain. Concerning the degree of post-operative pain, 14.6%, 39.6% and 38.2% had experienced mild, moderate and severe pain respectively. The mean of pain interference over the 24 hours in increasing order were: falling asleep (2±2.2), staying asleep (2.2±2.5), activities in bed (6.9±3), and activities out of bed (7.5±3.1). Pain caused patients to feel: anxious (2+2.5), depressed (2.4± 2.8), frightened (1.6±2.6) and helpless (0.9±1.7). Nausea, drowsiness, itching and dizziness were some of the side effects experienced by the patients when they received the anti-pain medications.

**Figure 1:**
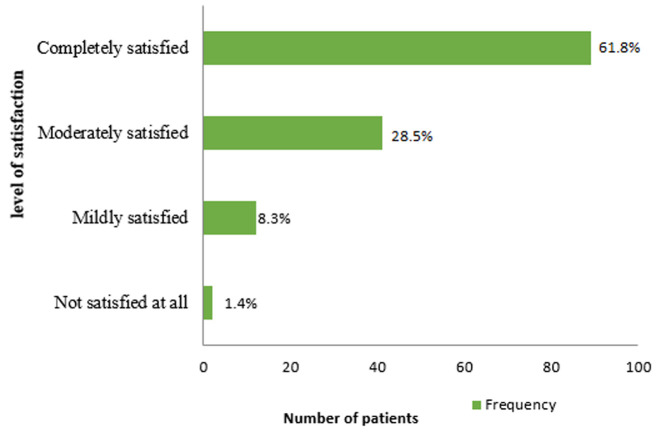
level of patients' satisfaction in Ayder Comprehensive Specialized Hospital from March 25^th^ to May 25^th^, 2017

**Patients' satisfaction with pain management and its predictors:** the level of patient satisfaction with their POP management in this study is presented in Figure 1. Over half of the patients (61.8%, n=89) were completely satisfied with their pain management protocols while 1.4% of the study participants were not satisfied at all. The overall mean satisfaction with pain relief management was 7.01±2.305 on the 0-10 numerical rating scale.

Stepwise linear regression analysis revealed that substance use, prior chronic pain, and prior surgical history were factors associated and substantiated to be predictors of patient satisfaction (F (3,140) =5.364, Adjusted R_2_= 0.084, P=0.02). Patients with prior chronic pain are more satisfied (B=0.190, p = 0.019) than those patients without prior chronic pain. Patients who use substance were more satisfied with their pain management protocols than those patients who did not use substance (B=0.216, P=0.008). On the other hand, patients with prior history of surgery are less satisfied compared with patients that did not have surgical history (B=-0.177, P=0.029) (Table 3).

**Table 3:** stepwise multiple linear regression model for patient characteristics and clinical outcomes as predictors of patient satisfaction from March 25^th^ to May 25^th^, 2017

Variables	Coefficients						
	Unstandardized		Standardized	T	P-value	95% CI for B	
	B	SE	B			Lower bound	Upper bound
Constant	6.381	0.322	------	19.789	0.000	5.744	7.019
Substance use	1.271	0.475	0.216	2.676	0.008	0.332	2.209
Prior chronic pain	0.898	0.379	0.190	2.369	0.019	0.148	1.65
Surgical history	-1.233	0.558	-0.177	-2.207	0.029	-2.337	-0.129

**Attitude and belief of patients on pain management:** the attitude and beliefs of patients on their pain management are summarized in Table 4. The indicators of barriers to adequate pain management from the patient side were adapted from the APSOPQ. Patients were asked to rate on a 0 to 5 scale (0: do not agree at all, 5: agree very much) about the level they agree with seven statements focused on common pain management misunderstandings. The higher ratings in the Likert scale indicate stronger attitude towards the myths which may lead to barriers (a hindering attitude) to achieving adequate pain management. For example, a strong belief that pain medication should be saved in case of worsening of the pain may mean a patient fails to request pain medication in a timely manner, which potentially leads to requiring higher doses in order to get pain under control. In this study, patients described a comparatively higher level of agreement on the Likert scale with all the seven statements, with the highest level of agreement regarding potential for addiction (3.11±1.609) and lowest level of agreement for the statement on control of pain by anti-pain medications (1.54±1.443).

**Table 4:** patients' attitudes and beliefs about pain management in Ayder Comprehensive Specialized Hospital from March 25^th^ to May 25^th^, 2017

Statement	Mean	SD
People get addicted to pain medication very easily	3.11	1.609
The experience of pain is sign that the illness has gotten worse	2.75	1.304
Pain medication should be ‘saved’ in case the pain gets worse	2.73	1.36
Complaints of pain could distract the doctor from treating my underlying illness	2.58	1.282
It is easier to put up with pain than with the side effects that come with pain treatments	2.44	1.413
Good patients avoid talking about pain	1.63	1.485
Pain medication cannot really control pain	1.54	1.443

## Discussion

Surgical patients in this study reported high levels of post-operative pain. Almost 92.4% of the patients studied had some sort of pain after 24 hours of surgery with 39.6% and 38.2% having moderate and severe pain respectively. Despite high reporting of moderate (61.8%) and severe pain (28.5%) the respondents were completely or moderately satisfied with their pain management respectively. This paradoxical high satisfaction of patients despite high reports of post-operative pain is supported by different literature. There is growing evidence of patient satisfaction with pain relief despite high pain score reports [14,17]. Thus, patient satisfaction may be irrelevant end point sometimes as far as pain management is concerned [18,19].

There was no significant difference in patients' satisfaction regardless of the variation in pain intensity level (One-way ANOVA, F (3,140) =1.609, P=0.190). This finding is consistent with previous studies [14]. However, a study done in the United States reported a negative correlation between pain intensity and patient satisfaction [20]. This difference might be due to the study design variation in which this study considers pain scores at discharge. In the current study, approximately 90% of the patients were moderately to completely satisfied with their post-operative pain management even though a substantial number of patients had moderate and severe level of pain score. This finding coincides with the results of studies done in Jimma University Hospital, Ethiopia and Nigeria [14,21] but, it was lower than other studies from developed and developing countries [22-24]. The reason for the contradictory high patient satisfaction in the face of high level of pain intensity might be explained due to: caring attitude of nurses and other health care professionals, existence of recurrent pain assessment, high rate of preoperative pain education, and a respectful communication atmosphere [24-26]. Nevertheless, in our study, the majority of participants reported they did not receive pain management education, and assistance on the use of non-pharmacological methods was minimal. Moreover, the result of this study showed there is a hindering attitude towards adequate pain management as described previously, which is in line with a previous study done in Jimma University Hospital [14].

The majority (58.3%) of the patients never requested any pain medication. This finding showed that patients of the current setting were less likely to request anti-pain medication in comparison to patients from other countries [27-29], even if they were suffering high levels of pain. This result may imply that patients had less involvement in their pain management and, due to high level of barriers; they are less likely to communicate their concerns and their needs to their healthcare providers. In the current study, the principal non-pharmacological means the study participants used to cope with their pain were listening music, praying, relaxation, and massage. This finding is different from the results of Chinese and Mexican patients who used self-prayer, intimate help, and altering their positions [28,30]. Large proportions (87.5%) of the patients were never encouraged by their healthcare professionals to use non-medicinal methods to control their pain, which may indicate a disjoint from medical care and patient needs. However, more sophisticated non-medicinal methods of pain relief like guided imagery and prayer by others which are usually practiced in American, Hispanic and Canadian populations were not readily available in our study setting [14,22,23].

This study demonstrated that substance use (B=0.216, p=0.008), prior chronic pain (B=0.19, p=0.019) and prior surgical history (B=-0.177, p=0.029) were strong predictors of patients' satisfaction with their pain management. This result is in contrast with the findings of some previous studies [15,24,26]. The reason for heterogeneous predictors of patients' satisfaction might be due to variation of study setting (hospital setting), knowledge, awareness and perception of patients towards pain and variation in the type of tool used. Given that the present study employed a cross-sectional study design, only the level of pain on the dates of the study was recorded and did not investigate the trends in of patients' pain over time. In addition, patients were not addressed pre-operatively to assess their preexisting pain, which may introduce recall bias. The merit of this study is that the questionnaire used was adapted from standard questionnaire and was the first study of this type done in the hospital.

## Conclusion

This study demonstrated a high level of patients' satisfaction with their pain management despite the majority of the patients complained of moderate and severe pain intensity. This finding indicates the need for future in-depth studies on this 'disconnect'. The study also showed the lack of inclusiveness of non-pharmaceutical interventions in the pain management strategies in the hospital. In part, this may reflect the lack of standard guidelines for pain management in this setting and for training of patients and health professionals on the merits of alternative approaches to post-operative pain management.

### What is known about this topic

- *There is paradoxically high level of patient satisfaction despite majority of the surgically treated patients experienced moderate to severe degree of pain;*
- *Sophisticated non-pharmacological pain treatment protocols are used especially in developed countries like USA and China*.

### What this study adds

- *This study determines the predictors of patient satisfaction i.e. substance use, prior chronic pain and prior surgical history;*
- *Non-pharmacological methods of pain management were found low in this study and this indicates there is a disjoint between the medical staff and the patient needs;*
- *This study reveals surgical patients had less involvement in their pain management and, due to high level of barriers and they are less likely to communicate their concerns and their needs to their healthcare providers*.
